# MYC‐activated lncRNA *HNF1A‐AS1* overexpression facilitates glioma progression via cooperating with *miR‐32‐5p*/*SOX4* axis

**DOI:** 10.1002/cam4.3186

**Published:** 2020-07-20

**Authors:** Jianheng Wu, Rong Li, Linfan Li, Yimian Gu, Hui Zhan, Changbao Zhou, Chuanhong Zhong

**Affiliations:** ^1^ Department of Neurosurgery Gaozhou People's Hospital Gaozhou Guangdong China; ^2^ Department of Radiation Oncology The First Affiliated Hospital Guangzhou Medical University Guangzhou Guangdong China; ^3^ Department of Neurosurgery The Affiliated Hospital of Southwest Medical University Neurosurgical Clinical Research Center of Sichuan Province Luzhou Sichuan China

**Keywords:** glioma, *HNF1A‐AS1*, *miR‐32‐5p*, *SOX4*

## Abstract

Mounting literatures have revealed the crucial effects of long noncoding RNA (lncRNA) in various cancers, including glioma. *HNF1A‐AS1*, a novel lncRNA, is reported to modulate tumorigenesis and development of multiple cancers. However, the tumorigenic function of lncRNA *HNF1A‐AS1* in glioma remains largely unknown. quantitative reverse transcription and polymerase chain reaction and western blot assays were applied to evaluate the expression of relevant mRNAs and proteins. 5‐Ethynyl‐2’‐ deoxyuridine, terminal deoxynucleotidyl transferase dUTP nick‐end labeling, flow cytometry, and transwell assays were conducted for examining the influence of *HNF1A‐AS1* on glioma cell functions. The relationship among RNAs was investigated by mechanical experiments. The results demonstrated that *HNF1A‐AS1* was predominantly highly expressed in glioma cell lines compared with nontumor glial epithelial cell, which was associated with the stimulation of transcription factor myelocytomatosis oncogene. Knockdown of *HNF1A‐AS1* remarkably inhibited glioma cells proliferation, migration, and invasion, while accelerating cell apoptosis in vitro. Mechanically, *HNF1A‐AS1* served as a *miR‐32‐5p* sponge. Moreover, *SOX4* was discovered as a target of *miR‐32‐5p*. Inhibited *miR‐32‐5p* or upregulated *SOX4* could markedly counteract the inhibitory effects of silencing *HNF1A‐AS1* on glioma malignant biological behaviors. *HNF1A‐AS1* exerted oncogenic property in glioma progression via upregulating *miR‐32‐5p*–mediated *SOX4* expression, suggesting potential novel therapeutic target for future glioma treatment.

## Graphical abstract

Silencing *HNF1A‐AS1* dampens glioma cell proliferation, migration, and invasion, while facilitating apoptosis. Myelocytomatosis oncogene induces overexpression of *HNF1A‐AS1* via transcription activation. *HNF1A‐AS1* directly binds to *miR‐32‐5p* and *miR‐32‐5p* inhibition could reverse the restraining effects of *HNF1A‐AS1* knockdown on glioma.

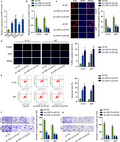

## 1 INTRODUCTION

Glioma is the most common intracranial malignant tumor and is one of the leading causes of cancer deaths.^1^ The occurrence of brain tumors is 21/100 000, which is much lower than other type of cancers, occupying only approximately 2% of all human cancers. However, the morbidity of glioma accounts for almost 60% among all kinds of brain tumors.^2^

Accumulating evidence highlighted the importance of abundantly transcribed noncoding transcripts, among which long noncoding RNAs (lncRNAs) emerged as critical mediator in many processes of cell biology. Recently, *HNF1A‐AS1*, as a novel identified lncRNA, gained much attention. *HNF1A‐AS1* is correlated with tumorigenic functions including cancer cell proliferation, migration, and invasion. For instance, *HNF1A‐AS1* was revealed to facilitate colon cancer metastatic progression via regulating miR‐34a/SIRT1/p53 feedback loop.^3^ Besides, overexpressed *HNF1A‐AS1* activates the Wnt/β‐catenin signaling pathway to promote colorectal cancer carcinogenesis.^4^

Substantial studies manifested that lncRNAs can function as competing endogenous RNAs (ceRNAs) in regulating the protein production of mRNA via binding to shared miRNAs post‐transcriptionally.^5^ This discovery deepened the understanding of human genome and highlighted the important gene regulatory role of lncRNAs, which were previously thought as “transcription noise”.^6^ Long noncoding RNAs could mediate various cellular biological functions including cell proliferation, apoptosis, migration, and invasion in pathology evolvement.^7^, ^8^ Increasing studies revealed the implication of lncRNAs in progression of brain tumors.^9^, ^10^ To date, the molecular relationship between lncRNA and glioma onset and progression has been unveiled. For instance, LINC00909 serves as a ceRNA to positively mediate the expression of MUC1‐C via binding to miR‐194, and consequently promotes glioma cell migration and invasion.^11^ Long noncoding RNA UBE2R2‐AS1 serves as a ceRNA against miR‐877‐3p to upregulate TLR4, and inhibits the malignant phenotype of glioblastoma cells.^12^ These studies indicated that lncRNAs are essential for glioma progression. Nevertheless, as a novel lncRNA, the biological role and molecular mechanisms of *HNF1A‐AS1* in glioma has not been explored yet. This study aimed to reveal the underlying molecular mechanism of *HNF1A‐AS1* in glioma progression.

## 2 MATERIALS AND METHODS

### 2.1 Tissue samples

In total, 35 glioma tissues and 10 normal tissues (excised from patients with other brain diseases) were obtained from Gaozhou People's Hospital with the approval from the Ethics Committee of Gaozhou People's Hospital. Each participant has signed informed consent. No patients have received chemo‐ or radiotherapy before surgery. After surgical resection, fresh tissues were instantly frozen in liquid nitrogen and stored at −80°C.

### 2.2 Cell lines

Human glioma cell lines (LN229, A172, SHG‐44, and U87) and normal brain glial cell line (HEB) were both procured from American Type Culture Collection (ATCC; Manassas, VA) and cultured under 95% air, 5% CO_2_ at 37°C. Dulbecco's Modified Eagle's medium (Invitrogen) adding 10% fetal bovine serum were applied for cell culture.

### 2.3 Quantitative reverse transcription and polymerase chain reaction (qRT‐PCR)

Total RNA from A172 and U87 cells were extracted by TRIzol reagent from Invitrogen in line with the guidebook. A Reverse Transcription Kit (Toyobo) was applied for synthesizing cDNA for conducting qRT‐PCR with SYBR Green Super Mix (Bio‐Rad). All RNA levels were measured via 2^−ΔΔCT^ method, with U6 or glyceraldehyde‐3‐phosphate dehydrogenase (GAPDH) as the internal reference.

### 2.4 Cell transfection

Cells of A172 and U87 were seeded in the 24‐well plates for 48 hours of transfection with Lipofectamine 2000 (Invitrogen). The duplex sh‐RNAs of *HNF1A‐AS1* (sh‐*HNF1A‐AS1*#1/2) and negative control (NC; termed short hairpin‐negative control [sh‐NC]), pcDNA3.1/myelocytomatosis oncogene (MYC), pcDNA3.1/*SOX4*, and NC (pcDNA3.1), as well as the *miR‐32‐5p* mimics and NC mimics, *miR‐32‐5p* inhibitor and NC inhibitor, all these were designed and synthesized by RiboBio.

### 2.5 5‐Ethynyl‐2’‐ deoxyuridine (EdU) staining

A172 and U87 cells on sterile coverslips were transfected and plated in the 24‐well plates for treatment with the EdU incorporation assay kit (Ribobio). Cell nucleus was counterstained with EdU and DAPI (Beyotime), and visualized with laser confocal microscopy (Olympus).

### 2.6 Terminal deoxynucleotidyl transferase dUTP nick‐end labeling assay

Glioma cells after transfection were cultured with the terminal deoxynucleotidyl transferase dUTP nick‐end labeling (TUNEL) Apoptosis Assay Kit (Beyotime) in light of the user guide. After treating with DAPI, apoptotic cells were analyzed under confocal microscopy.

### 2.7 Flow cytometry of apoptosis

70% cold ethanol on ice was used to fix transfected glioma cells for 1 hour. Then, cells were treated with Annexin V‐APC and 7‐AAD staining in the dark. A flow cytometer (BD Biosciences) was used to assess cell apoptosis.

### 2.8 Transwell assay

Transfected cells were reaped and planted into the upper chamber of transwell inserts (Corning Incorporated) coating with or without Matrigel membrane (BD Biosciences) for invasion or migration analysis. After 48 hours, the invaded and migrated cells were stained in crystal violet. Then, a microscope (magnification, ×200) was used to count the cells.

### 2.9 Chromatin immunoprecipitation assay

Cells of A172 and U87 were fixed for 10 minutes to generate DNA‐protein cross‐links. After shearing into 200‐ to 1000‐bp chromatin fragments, cell lysates were immunoprecipitated with anti‐MYC or normal control anti‐immunoglobulin G (IgG) antibody (Millipore). The retrieved chromatin DNA by beads was subjected to qRT‐PCR analysis.

### 2.10 Dual‐luciferase reporter assays

HEK‐293T cells (ATCC) in the 24‐well plates were co‐transfected with the pGL3 vector containing *HNF1A‐AS1* promoter, pRL‐TK‐Renilla plasmid (Promega Corporation), and pcDNA3.1/MYC or pcDNA3.1. Besides, the wild‐type (WT) or mutant (MUT) reporter constructs of *HNF1A‐AS1* and *SOX4* were generated by pmirGLO vectors and co‐transfected into HEK‐293T cells with *miR‐32‐5p* mimics and NC mimics. The Dual‐Luciferase Reporter Assay System (Promega) was employed to evaluate luciferase intensity after 48 hours of transfection.

### 2.11 Subcellular fractionation

A PARIS™ Kit (Invitrogen) was applied for isolating nuclear and cytoplasmic RNAs in 1 × 10^6^ glioma cells, which were previously rinsed in precooled PBS. The isolated *HNF1A‐AS1* was analyzed via qRT‐PCR. U6 and GAPDH, respectively, served as the nuclear and cytoplasmic controls.

### 2.12 Fluorescence in situ hybridization assay

After fixation, the glioma cells were collected for incubation with RNA Fluorescence in situ hybridization (FISH) probe for *HNF1A‐AS1* (RiboBio) in the hybridization buffer. After slides were cultured with DAPI solution, cells were observed under a Olympus microscope.

### 2.13 RNA pull down

The protein extracts from glioma cells were obtained for mixing with biotin‐labeled *HNF1A‐AS1* (Biotin *HNF1A‐AS1* WT/MUT) and Biotin NC, as well as the beads for 1 hour. qRT‐PCR was followed for analyzing the mixture of pull‐down.

### 2.14 RNA immunoprecipitation assay

1 × 10^7^ glioma cells in RNA immunoprecipitation lysis buffer were collected and immunoprecipitated with anti‐Ago2 or normal mouse control anti‐IgG antibody (Millipore). Finally, the purified RNA was analyzed by qRT‐PCR.

### 2.15 Western blot

The cellular protein samples from glioma cells were prepared for separation with SDS‐PAGE (10%) and electrotransferred to PVDF membranes. After culturing with 5% skimmed milk, membranes were incubated with primary antibodies (Abcam) against *SOX4* and control GAPDH all night, then with horseradish peroxidase‐tagged secondary antibody for 2 hours. The band intensity was determined by enhanced chemiluminescence reagent (Santa Cruz Biotechnology).

### 2.16 Animal study

Six‐week‐old male nude mice was maintained in the SPF‐grade animal laboratory, acquired from the National Laboratory Animal Center (Beijing, China). All procedures were approved by the Animal Research Ethics Committee of Gaozhou People's Hospital. Animal study was achieved through subcutaneous inoculation of 5 × 10^6^ A172 cells to mice for 4 weeks. Tumor volume was recorded every 4 days. After killing mice, tumors were excised and weighed for analysis.

### 2.17 Statistical analysis

All data were analyzed statistically via a *t* test or one‐way ANOVA by using PRISM 6 (GraphPad), with *P* < .05 as significant level. Results were given as the mean ± SD of three or more repetitive assays.

## 3 RESULTS

### 3.1 Silencing *HNF1A‐AS1* dampens glioma cell proliferation, migration, and invasion, while facilitating apoptosis

**FIGURE 1:**
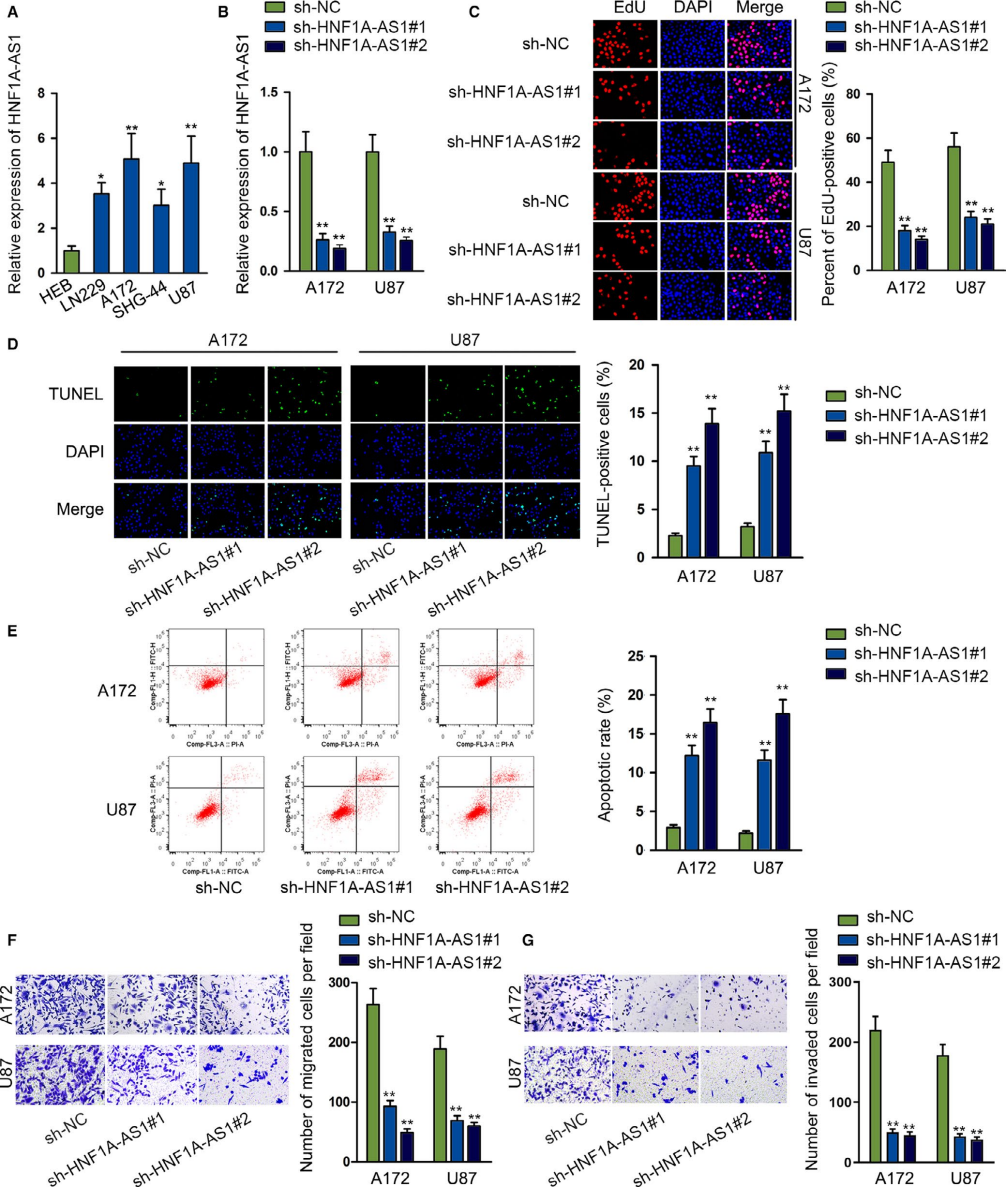
Silencing *HNF1A‐AS1* dampens glioma cells proliferation, migration, and invasion, while facilitating apoptosis. A, qRT‐PCR analysis was used to determine the expression profile of *HNF1A‐AS1* in glioma cell lines and normal brain glial cell. B, The transfection efficiency of sh‐*HNF1A‐AS1*#1 and sh‐*HNF1A‐AS1*#2 were detected by qRT‐PCR analysis. C, EdU assay was performed to assess glioma cells proliferation ability. D and E, TUNEL and flow cytometry assays were performed to determine cell apoptosis. F and G, Transwell migration and invasion assays were conducted to assess glioma cells migration and invasion ability. TUNEL, terminal deoxynucleotidyl transferase dUTP nick‐end labeling. *P < .05, **P < .01

To understand the expression status of *HNF1A‐AS1* in glioma, we performed qRT‐PCR. It was revealed that *HNF1A‐AS1* was significantly upregulated in glioma tissues than in normal tissues (Figure S1A). We also noticed that *HNF1A‐AS1* was significantly overexpressed in glioma cells (LN229, A172, SHG‐44, and U87) in contrast to normal brain glial cell line (HEB) (Figure 1A). We also noticed that A172 and U87 cells contained the most significant upregulation of *HNF1A‐AS1*; thus they were used for following assays. For loss‐of‐function assays, we utilized two sh‐RNAs directly targeting *HNF1A‐AS1* and verified the inhibitory efficiency through qRT‐PCR (Figure 1B). The EdU assay showed that silencing *HNF1A‐AS1* significantly inhibited cell proliferation, as illustrated in Figure 1C. The TUNEL assay indicated that *HNF1A‐AS1* knockdown could markedly promote glioma cell apoptosis (Figure 1D). Flow cytometry data showed notably elevated cell apoptosis after silencing *HNF1A‐AS1*, which further confirmed the pro‐apoptosis effects of *HNF1A‐AS1* knockdown (Figure 1E). Besides, silencing *HNF1A‐AS1* could significantly restrain cell migration and invasion, as shown in transwell assays (Figure 1F,G). Together, *HNF1A‐AS1* played an oncogenic role in glioma progression.

### 3.2 MYC induces overexpression of *HNF1A‐AS1* via transcription activation

**FIGURE 2:**
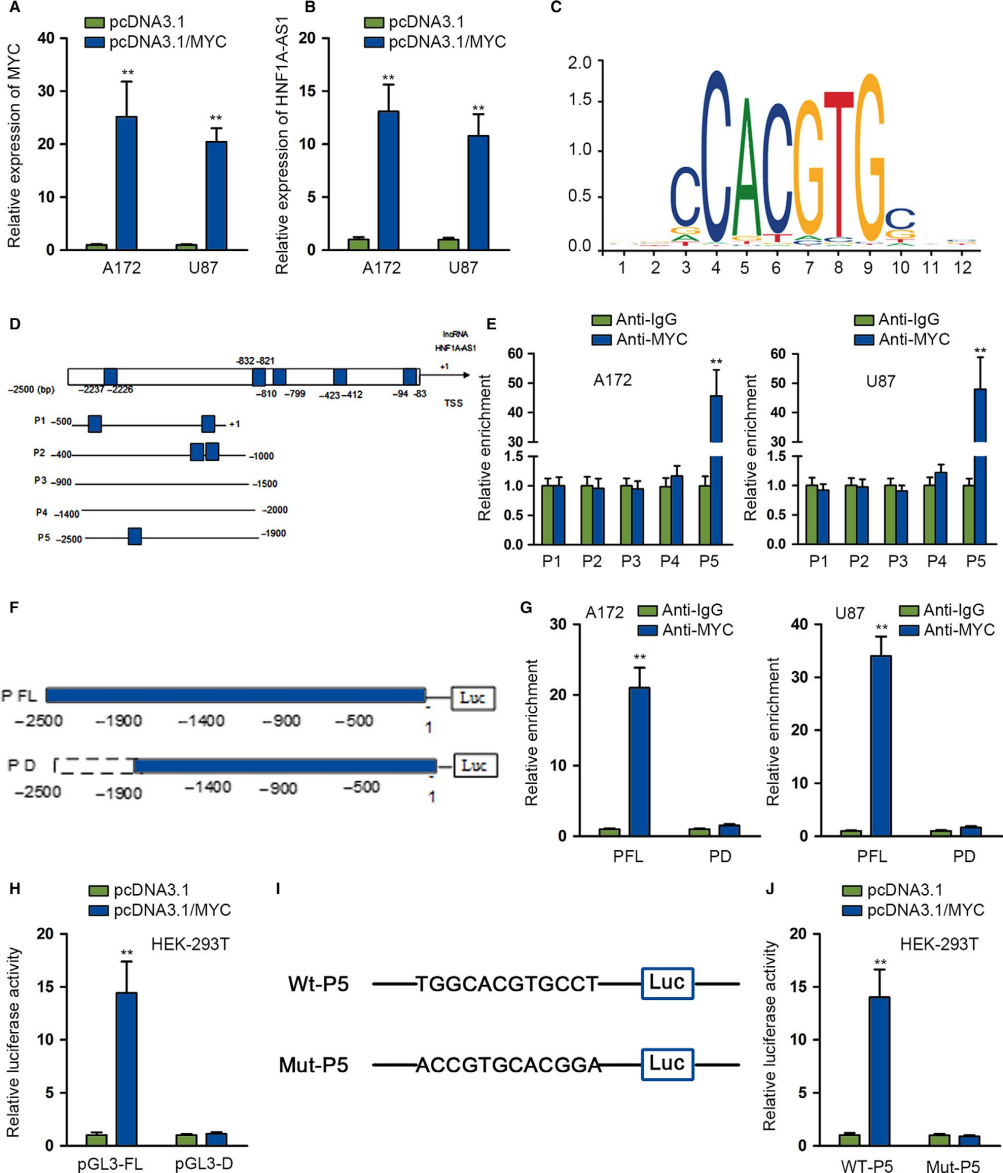
MYC induces overexpression of *HNF1A‐AS1* via transcription activation. The expression of MYC was determined by quantitative RT‐PCR (qRT‐PCR) analysis after transfecting with pcDNA3.1 and pcDNA3.1/MYC plasmids. B, qRT‐PCR was performed to study the effects of overexpressing MYC on *HNF1A‐AS1* expression. C, The putative MYC binding motif in human *HNF1A‐AS1* promoter. D, *HNF1A‐AS1* promoter was divided into four sections according to potential MYC binding sites. E, Chromatin immunoprecipitation (ChIP) assay using antibody targeting MYC and IgG was conducted to detect the affinity of MYC with *HNF1A‐AS1* promoter. F, The full *HNF1A‐AS1* promoter (P FL) and *HNF1A‐AS1* P5 deleted (P D) were subcloned into pGL3 luciferase reporter vector. G, ChIP assay was used to investigate the relative enrichment of *HNF1A‐AS1* promoter. H, Luciferase reporter assay was performed in HEK‐293T. I, pGL3‐WT‐P5, pGL3‐MUT‐P5 luciferase vectors were constructed accordingly. J, HEK‐293T was co‐transfected with pcDNA3.1 and pcDNA3.1/MYC and a luciferase reporter was performed between WT‐P5 and MUT‐P5. **P < .01

We hypothesized that transcription factor might be associated with *HNF1A‐AS1* overexpression in glioma cells. MYC might bind to *HNF1A‐AS1* promoter region by prediction of UCSC (http://genome.ucsc.edu/) and Jaspar (http://jaspardev.genereg.net/) database jointly. The influence of MYC on *HNF1A‐AS1* expression was subsequently detected. We observed that pcDNA3.1/MYC could force the overexpression of MYC efficiently (Figure 2A). We noticed that *HNF1A‐AS1* expression was elevated after overexpressing MYC (Figure 2B). With the aid of Jaspar, we detected the putative MYC motif in human *HNF1A‐AS1* promoter region, as shown on Figure 2C. Then, we found five concrete putative MYC binding sequences in *HNF1A‐AS1* promoter by Jaspar. We divided the promoter region into five sectional area from −2500 bp according to putative binding sites predicted by Jaspar (Figure 2D). Then, the binding capacity of MYC to *HNF1A‐AS1* promoter was investigated by chromatin immunoprecipitation (ChIP) assay. Its findings depicted that MYC bound to P5 section (Figure 2E). Moreover, we constructed pGL3 luciferase vector containing *HNF1A‐AS1* full promoter region (P FL) and *HNF1A‐AS1* promoter P5 deleted region (P D) (Figure 2F). Deletion of P5 abrogated the increased promoter enrichment in anti‐MYC, indicating that P5 fragment in *HNF1A‐AS1* promoter region was indispensable for the interaction of *HNF1A‐AS1* promoter with MYC (Figure 2G). This phenomenon was further elucidated by luciferase reporter assay on HEK‐293T cell. It indicated that pcDNA3.1/MYC elevated the promoter activity of pGL3‐FL, not that of pGL3‐D (Figure 2H). These data represented that MYC interacted with *HNF1A‐AS1* promoter at approximately −2500 to −1900 bp upstream transcription start site (TSS). We found that there was only one specific binding site (−2237 to −2226) predicted by Jaspar in P5 fragment. Therefore, we intended to verify whether MYC bound to these sequences in P5 segment. We constructed P5‐WT and P5‐MUT (which mutated −2237 to −2226 sequence) accordingly (Figure 2I). Promoter activity of P5‐WT was strengthened after overexpressing MYC, while P5‐MUT, which lacked the sequence form −2237 to −2226, showed no alternation in luciferase activity after co‐transfection with pcDNA3.1/MYC (Figure 2J). Collectively, these data provided substantial proof for the activation of *HNF1A‐AS1* transcription activity by MYC in glioma cells.

### 3.3 *HNF1A‐AS1* directly binds to *miR‐32‐5p* and *miR‐32‐5p* inhibition could reverse the restraining effects of *HNF1A‐AS1* knockdown on glioma

**FIGURE 3:**
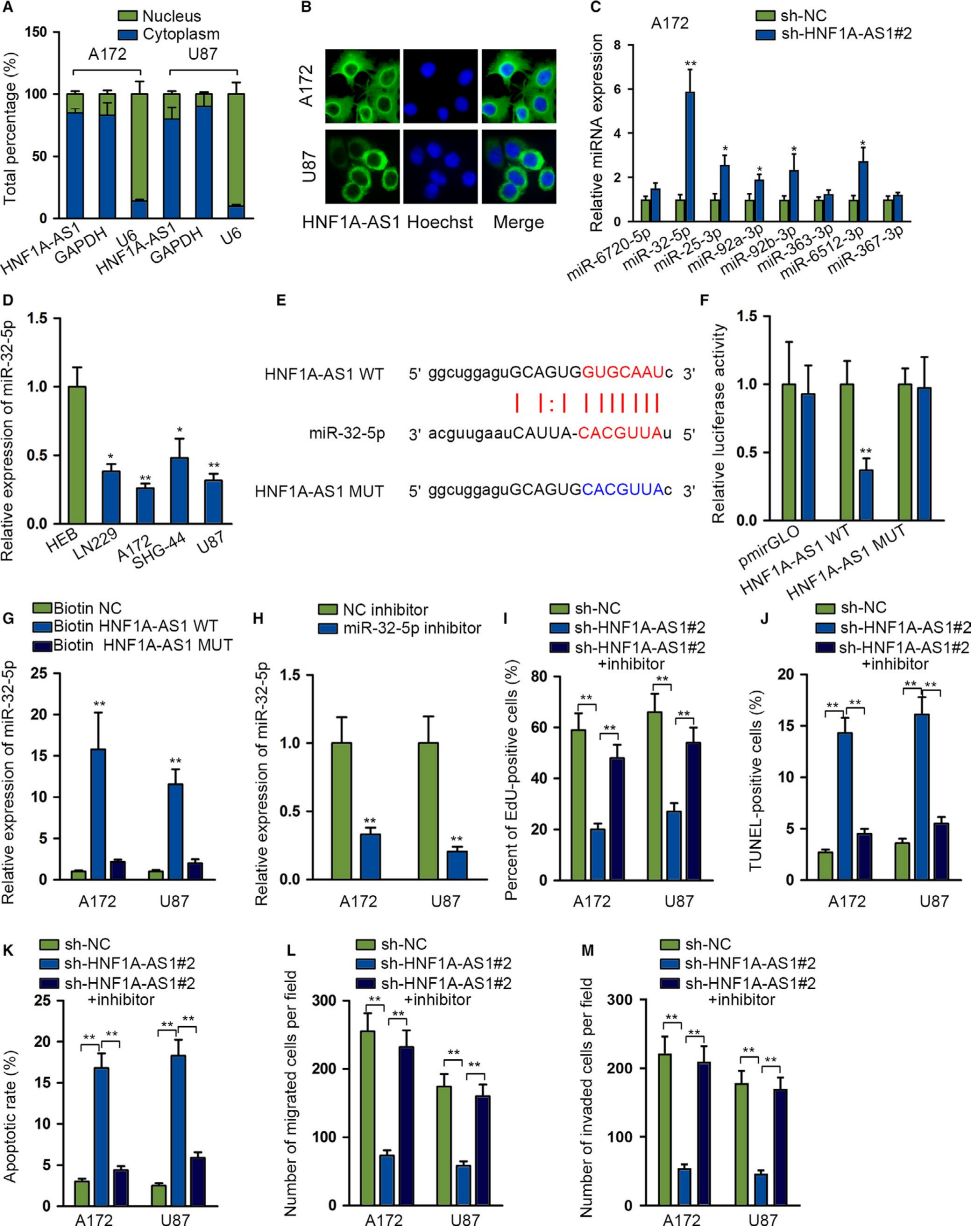
*HNF1A‐AS1* directly binds to *miR‐32‐5p* and *miR‐32‐5p* inhibition could reverse the restraining effects of *HNF1A‐AS1* knockdown on glioma. A and B, Subcellular fractionation and FISH assays were performed to determine the location of *HNF1A‐AS1*. C, Quantitative RT‐PCR (qRT‐PCR) analysis was performed to determine miRNAs expression after *HNF1A‐AS1* depletion. D, The expression status of *miR‐32‐5p* was detected in glioma cell lines and normal brain glial cell lines. E, Putative wild and mutant *miR‐32‐5p* binding sites with *HNF1A‐AS1*. F, Luciferase reporters were performed in HEK‐293T. MiR‐32‐5p mimics could impair the luciferase activity of *HNF1A‐AS1*‐WT. G, RNA pull down showed enriched *miR‐32‐5p* by biotin‐labeled *HNF1A‐AS1*‐WT. H, qRT‐PCR was used to examine the transfection efficiency of *miR‐32‐5p* inhibitor. I‐M, Functional experiments were conducted in A172 and U87 cell lines to examine the rescue effects of silenced *miR‐32‐5p* on silenced *HNF1A‐AS1* in cell proliferation, apoptosis, migration, and invasion. *P < .05, **P < .01

Many cytoplasmic lncRNAs have been found as ceRNAs in the carcinogenesis of cancers. *HNF1A‐AS1* was found mainly situated in cytoplasm through subcellular fraction and FISH assays, as illustrated in Figure 3A,B. By browsing starBase tool,^13^ eight miRNAs were found to interact with *HNF1A‐AS1* (clip data: low stringency ≥ 1). Among them, *miR‐32‐5p* was upregulated significantly in A172 after silencing *HNF1A‐AS1* compared with other miRNA candidates from qRT‐PCR results (Figure 3C). Subsequently, we detected an abnormal low expression status of *miR‐32‐5p* in glioma cells (Figure 3D). By browsing starBase, putative *miR‐32‐5p* binding site with *HNF1A‐AS1* was detected. We mutated the putative binding site sequence correspondingly, as shown in Figure 3E. Both *HNF1A‐AS1*‐WT and *HNF1A‐AS1*‐MUT were subcloned into pmirGLO luciferase reporter vector. After co‐transfecting *miR‐32‐5p* mimics with pmirGLO vector containing *HNF1A‐AS1*‐WT or *HNF1A‐AS1*‐MUT, the luciferase activity of *HNF1A‐AS1*‐WT was markedly attenuated, instead of *HNF1A‐AS1*‐MUT in HEK‐293T, as illustrated in Figure 3F. MiR‐32‐5p was only significantly pulled down by Bio‐*HNF1A‐AS1*‐WT group, which verified the interaction between *HNF1A‐AS1* and *miR‐32‐5p* (Figure 3G). *HNF1A‐AS1* bound with *miR‐32‐5p* and negatively modulated the expression of *miR‐32‐5p* in glioma cells.

After that, we carried out rescue assays to determine whether *HNF1A‐AS1* contributed to glioma progression via inhibiting *miR‐32‐5p*. MiR‐32‐5p expression was silenced via transfection of plasmids containing *miR‐32‐5p* inhibitor into A172 and U87 cell lines (Figure 3H). MiR‐32‐5p inhibition obviously restored the oncogenic effects of *HNF1A‐AS1* in glioma cell proliferation (Figure 3I), apoptosis (Figure 3J,K; Figure S1B), migration (Figure 3L; Figure S1C), and invasion (Figure 3M; Figure S1D). These results elucidated that *HNF1A‐AS1* mediated glioma development via downregulating *miR‐32‐5p*.

### 3.4 *SOX4* is a direct target gene of *miR‐32‐5p*

**FIGURE 4:**
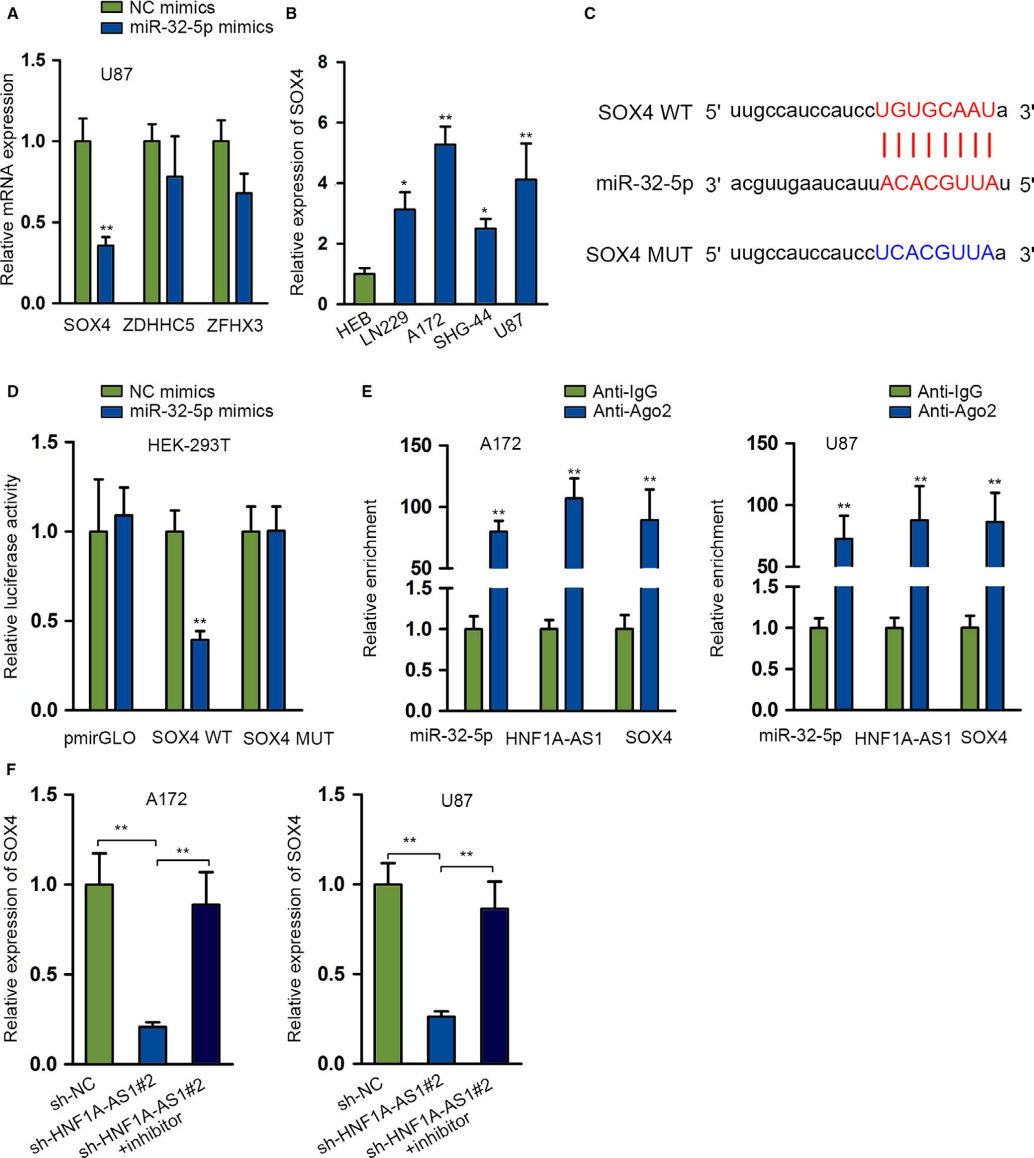
*SOX4* is a direct target gene of *miR‐32‐5p*. A, The expression of potential target genes was detected by quantitative RT‐PCR (qRT‐PCR) after transfecting *miR‐32‐5p* mimics. B, The expression profile of *SOX4* in cell lines was detected by qRT‐PCR. C, Putative wild and mutant *miR‐32‐5p* binding sites in 3ʹUTR sequence of *SOX4* based on starBase data. D. Luciferase reporter assays were performed to investigate the association between *miR‐32‐5p* and *SOX4*. E, RIP was used performed to verify the mechanical relationship among *miR‐32‐5p*, *HNF1A‐AS1* and *SOX4*. F. The effects of silenced *miR‐32‐5p* on silenced *HNF1A‐AS1* in *SOX4* expression were studied by qRT‐PCR. *P < .05, **P < .01

We aimed to find mRNA targets of *miR‐32‐5p* to better understand its role in glioma. Aided by starBase, we screened three target genes (*SOX4*, ZDHHC5, ZFHX3) according to below circumstance (clip data: strict stringency ≥ 5; degradome data: high stringency ≥ 3; program number: five; predicted program: microT, miRanda, miRmap, PITA, PicTar). We found that *SOX4* was significantly downregulated in the presence of *miR‐32‐5p* mimics compared with the other two mRNA candidates (Figure 4A). From qRT‐PCR results, we found a significant elevation of *SOX4* in four glioma cells (Figure 4B). This expression status is contrary to that of *miR‐32‐5p* in glioma cells, thus we chose *SOX4* to study. The putative *miR‐32‐5p* binding site in the 3′‐untranslated region (3ʹUTR) sequence of *SOX4* (Figure 4C) was verified by the results of dual luciferase reporter assays, which showed significant decreased luciferase activity only in *SOX4*‐WT by *miR‐32‐5p* upregulation (Figure 4D). RNA immunoprecipitation assay data showed significant enriched *miR‐32‐5p*, *HNF1A‐AS1*, *SOX4* in the anti‐Ago2 group instead of the IgG group (Figure 4E). The expression of *SOX4* was examined after silencing *HNF1A‐AS1* and then inhibiting *miR‐32‐5p* in A172 and U87. It was manifested that *SOX4* was downregulated after silencing *HNF1A‐AS1*, yet increased again in the presence of *miR‐32‐5p* inhibition (Figure 4F). Together, these results confirmed the ceRNA role of *HNF1A‐AS1* in glioma via regulating *miR‐32‐5p*/*SOX4* axis.

### 3.5 *SOX4* enrichment restores the carcinogenesis role of *HNF1A‐AS1*

**FIGURE 5:**
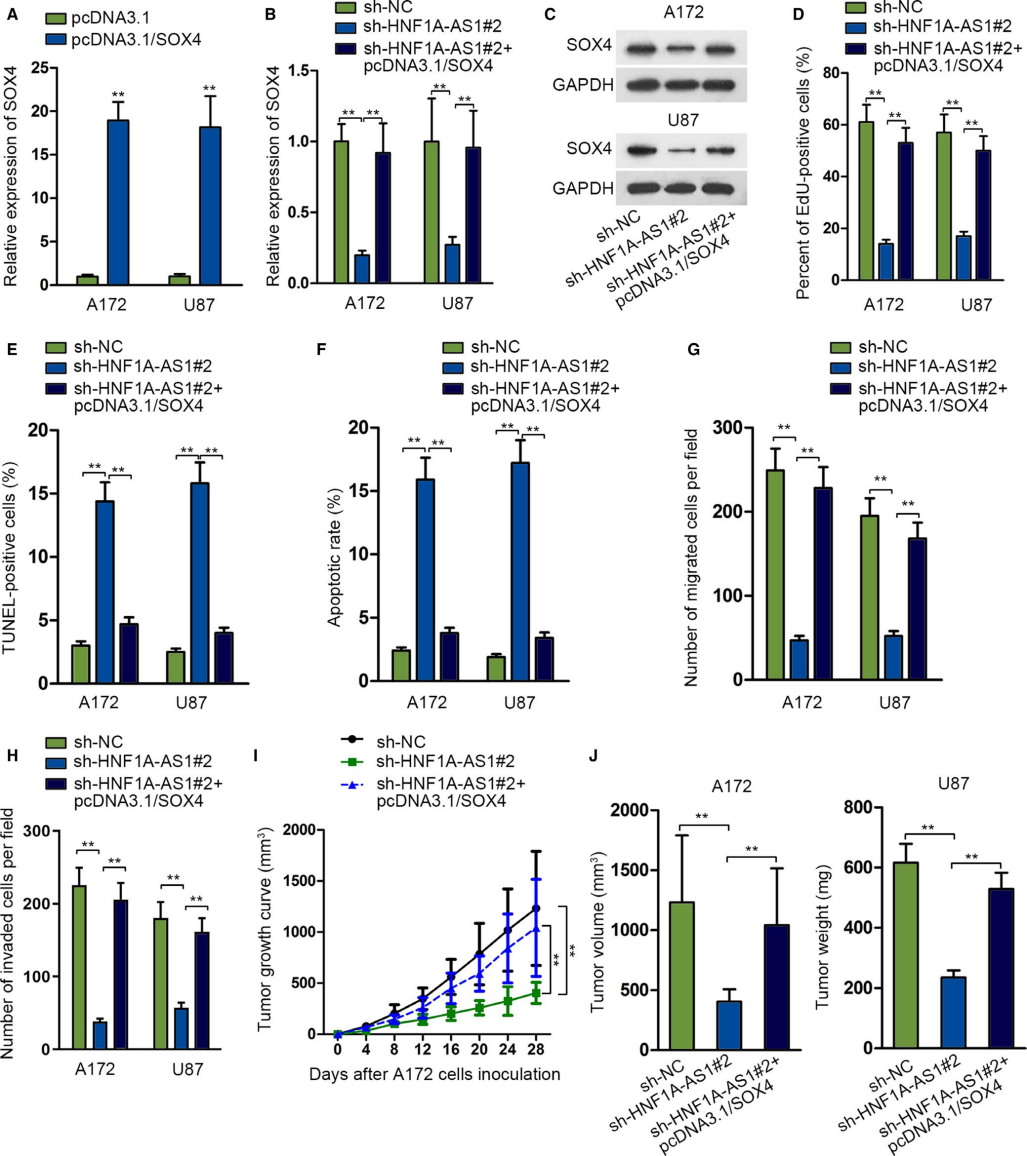
*SOX4* enrichment restores the carcinogenesis role of *HNF1A‐AS1*. A, The expression of *SOX4* after transfecting with pcDNA3.1/*SOX4* was studied through quantitative RT‐PCR (qRT‐PCR) assay. B‐C, qRT‐PCR and western blot assays were used to detect the expression of *SOX4* mRNA and protein expression level. D‐H, Rescue functional experiments were conducted among sh‐NC, sh‐*HNF1A‐AS1*#2, and sh‐*HNF1A‐AS1*#2 + pcDNA3.1/*SOX4* groups in A172 and U87 cells. I‐K, Xenograft tumor growth curve, volume, and weight were compared among sh‐NC, sh‐*HNF1A‐AS1*#2, and sh‐*HNF1A‐AS1*#2 + pcDNA3.1/*SOX4* groups. **P < .01

To further determine whether *HNF1A‐AS1* facilitated glioma via acting as a ceRNA in regulating *miR‐32‐5p*/*SOX4* network, we performed rescue experiments functionally. First, we validated the overexpression efficiency of *SOX4* (Figure 5A). Next, we co‐transfected pcDNA3.1/*SOX4* into *HNF1A‐AS1*–depleted A172 and U87 cell lines. The mRNA and protein levels of *SOX4* were inhibited after silencing *HNF1A‐AS1*, while recovered again after co‐transfecting with pcDNA3.1/*SOX4* form qRT‐PCR and western blot assays (Figure 5B,C). Based on the findings of functional rescue experiments, *SOX4* upregulation could reverse *HNF1A‐AS1* silencing produced biological effects on cell proliferation (Figure 5D), apoptosis (Figure 5E,F; Figure S1E), migration (Figure 5G; Figure S1F), and invasion (Figure 5H; Figure S1G). Furthermore, we constructed xenograft mice models by inoculating A172 cell lines transfected with sh‐NC, sh‐*HNF1A‐AS1*#2, and sh‐*HNF1A‐AS1*#2 + pcDNA3.1/*SOX4*. We found that silencing *HNF1A‐AS1* could suppress tumor growth, but this effect was reversed by overexpressing *SOX4* (Figure 5I; Figure S1H). Tumor volume and weight were reduced by silenced *HNF1A‐AS1* while co‐transfection of *SOX4* rescued the suppressive influence of silenced *HNF1A‐AS1* on tumor volume and weight (Figure 5J,K). To sum up, *HNF1A‐AS1* contributed to glioma progression by targeting *miR‐32‐5p*/*SOX4* axis.

## 4 DISCUSSION

Glioma is one of the most common types of intracranial tumors which initiate from the central nervous system. The prognosis of patients with high‐grade glioma is much worse than those with low‐grade glioma.^14^ Substantial studies supported the crucial regulator roles of lncRNAs in the initiation, development, and progression of various diseases, including tumors.^15^, ^16^, ^17^ *HNF1A‐AS1* has been reported to exhibit carcinogenesis property in gastric cancer^18^ and non–small cell lung cancer.^19^ In the present study, we found the aberrant overexpression of *HNF1A‐AS1* in glioma cell lines, which was in accordance with *HNF1A‐AS1* overexpression in urothelial carcinoma of the bladder.^20^ We noticed that the overexpression of *HNF1A‐AS1* was activated by transcription factor MYC. Previously, MYC amplification has been discovered to promote homologous recombination via targeting CDK18 in glioblastoma.^21^ We found that *HNF1A‐AS1* knockdown could suppress glioma cells proliferation, migration, and invasion abilities, while markedly enhance apoptosis capacity, which revealed the crucial oncogenic role of *HNF1A‐AS1* in glioma progression. These findings accord with the function of *HNF1A‐AS1* in oral squamous cell carcinoma.^22^

Over the last decade, the ceRNA crosstalk is prevalent. Numerous reports have found that a large number of lncRNAs were involved in the ceRNA network.^23^ LncRNAs could mediate the protein production of genes via acting as ceRNA according to substantial documents. Here, we detected the cytoplasmic localization of *HNF1A‐AS1* through subcellular fractionation and FISH assays. We screened downstream combinable miRNAs with *HNF1A‐AS1* and selected *miR‐32‐5p* through multiple channel. Inhibited *miR‐32‐5p* counteracted the curbing influence of inhibited *HNF1A‐AS1* in glioma progression. We initially uncovered that *HNF1A‐AS1* bound with *miR‐32‐5p* and decreased its expression in glioma cells. MiR‐32‐5p also presents low expression and involve in the ceRNA axis of SNHG14/*miR‐32‐5p*/SKIL in colorectal cancer.^24^ MiR‐32‐5p targets HOXB8 to repress the cellular malignant behavior in cervical cancer cells.^25^ LncRNA GAS5 modulates *miR‐32‐5p*/PTEN axis to suppress pancreatic cancer metastasis.^26^

Subsequently, we screened *SOX4* as the most potential target gene of *miR‐32‐5p* with putative binding site in its 3ʹUTR sequences. *SOX4* has been reported to be an oncogene supported by multiple lines of evidence. *SOX4* gene has been reported to be frequently amplified and upregulated in over 20 types of malignant tumors.^27^, ^28^ In the field of glioma, *SOX4* has been found to be significantly elevated in glioblastoma multiforme.^29^ Herein, we also detected the abnormal upregulation of *SOX4* in glioma cells in comparison to normal control cell, which was the same expression profile previously revealed in medulloblastoma.^30^ We clarified the oncogenic role of SXO2 in glioma via a collection of functional experiments. These data showed that *SOX4* overexpression could dampen cells proliferation, migration, and invasion, while stimulating apoptosis in glioma cells. Last but not least, *SOX4* overexpression could significantly reduce the biological effects induced by silencing *HNF1A‐AS1* in glioma cells, which further confirmed the ceRNA role of *SOX4*.

All in all, we initially found that MYC induced *HNF1A‐AS1* overexpression promoted glioma progression via modulating *miR‐32‐5p*/*SOX4* axis, providing potent reference value for targeting *HNF1A‐AS1* in future therapeutic strategies of glioma.

## CONFLICTS OF INTERESTS

None.

## AUTHORS' CONTRIBUTIONS

Jianheng Wu designed this study. Rong Li interpreted data. Linfan Li recorded data. Yimian Gu and Hui Zhan were responsible for preparation and investigaton. Jianheng Wu, Rong Li and Changbao Zhou devoted to data curation and methods. Chuanhong Zhong wrote the manuscript. All authors reviewed the manuscript.

## ETHICAL APPROVAL

All procedures were approved by the Ethics Committee of Gaozhou People's Hospital.

## Supporting information

Figure S1

## Data Availability

Research data and material are not shared.
