# Supplementary material for: MYC‐activated lncRNA HNF1A‐AS1 overexpression facilitates glioma progression via cooperating with miR‐32‐5p/SOX4 axis
Source: Cancer Med. 2020 Jul 20;9(17):6387–98. doi: 10.1002/cam4.3186 (PMC7476832; doi:10.1002/cam4.3186)
Supplement: Supplementary file 1 — Figure S1 [file CAM4-9-6387-s001.docx]

**Figure S1.** A. Relative expression of HNF1A-AS1 in 35 glioma tissues and 10 normal tissues were revealed by qRT-PCR assay. B-D. Original figures of flow cytometry analysis, transwell migration, and invasion assays for Figure 3K, 3L, 3M. E-G. Original figures of flow cytometry analysis, transwell migration and invasion assays for Figure 5F, 5G, 5H. H. Picture of xenograft tumors. **P < 0.01.
